# Fecal microbial composition associated with testosterone in the development of Meishan male pigs

**DOI:** 10.3389/fmicb.2023.1257295

**Published:** 2023-11-20

**Authors:** Xueyuan Jiang, Shaoshan Deng, Naisheng Lu, Wen Yao, Dong Xia, Weilong Tu, Hulong Lei, Peng Jia, Yeqing Gan

**Affiliations:** ^1^Shanghai Engineering Research Center of Breeding Pig, Livestock and Poultry Resources (Pig) Evaluation and Utilization Key Laboratory of Ministry of Agriculture and Rural Affairs, Institute of Animal Husbandry and Veterinary Science, Shanghai Academy of Agricultural Sciences, Shanghai, China; ^2^College of Animal Science and Technology, Nanjing Agricultural University, Nanjing, China; ^3^Meishan Pig Breeding Center of Jiading, Shanghai, China

**Keywords:** Meishan male pigs, testosterone, microbiome, development, interaction

## Abstract

**Introduction:**

The gut microbiota closely relates to host health, whereas the relationship between gut microbiota and testosterone during the development of Meishan male pigs remains unclear. This study investigated the fecal microbiota composition and testosterone level during development in Meishan male pigs.

**Methods:**

Fresh fecal samples of 20 healthy Meishan male pigs were individually collected at 10 and 22 weeks (wk) of age for testosterone content detection and bacteria pyrosequencing analysis. Anaerobic culture experiment of fecal bacteria in vitro was performed for bacteria pyrosequencing analysis.

**Results:**

The fecal testosterone content increased significantly from 10 weeks (wk) to 22 wk of age (*P* < 0.05). Meanwhile, the boars at 22 wk had a lower abundance of phylum *Bacteroidetes* and *Proteobacteria*, and genus *Alloprevotella*, *Prevotella_1*, *Prevotellaceae_NK3B31_group*, and *Streptococcus* in the fecal microbiota composition (*P* < 0.05). but higher proportions of the phylum *Actinobacteria*, *Firmicutes*, *Kiritimatiellaeota*, and *Tenericutes*, and genus *Clostridium_sensu_stricto_1*, *Muribaculaceae* and *Terrisporobacter* than that at 10 wk (*P* < 0.05), and the Firmicutes to Bacteroidetes ratio was higher at 22 wk than 10 wk (*P* < 0.05). Moreover, the fecal testosterone level significantly correlated with the relative abundance of the phylum *Actinobacteria*, *Firmicutes*, and *Tencuteseri*, and genus *Alloprevotella*, *Clostridium_sensu_stricto_1*, *Muribaculaceae*, *Prevotella_1* and *Streptococcus*. Furthermore, the *in vitro* experiments indicated that the abundance of the phylum *Proteobacteria* and genus *Escherichia-Shigella* reduced with the increase of supplemental testosterone level. In contrast, the proportion of *Firmicutes* phylum increased with additional testosterone levels.

**Discussion:**

Testosterone could modulate the microflora structure. Meanwhile, the bacteria could degrade the testosterone in a dose testosterone-dependent manner. These results provide us with new insights into the relationship between the gut microbiome and testosterone and the contributions of the gut microbiome in physiological regulation in response to gonad development.

## 1 Introduction

It is well known that the gut microbiome plays a vital role in the health status of the host. Describing the complexity and changes of the intestinal microbiota may help understand its effects on overall animal health. Moreover, the gut microbiome is regulated by many factors, including environmental variables, host genetics, and even steroid hormones. For instance, cortisol increases the proliferation of *Salmonella* in primary porcine alveolar macrophages (Verbrugghe et al., 2011). Mudd et al. (2017) observed that the fecal *Ruminococcus* population negatively correlated to the serum cortisol level in piglets. Experiments on weaning piglets showed a significant correlation between elevated cortisol levels and pathogenic bacteria at weaning (Moeser et al., 2007; Tuchscherer et al., 2018). Estradiol and progesterone inhibited the growth of *Helicobacter pylori in vitro* culture (Hosoda et al., 2011). Progesterone could increase the relative abundance of the *Bifidobacterium* genus in the gut microbial composition (Nuriel-Ohayon et al., 2019). All these studies suggest that steroid hormones may influence the proliferation and metabolism of bacteria.

Testosterone is a steroid hormone secreted from the Leydig cells of the testes in males or the ovaries of females, and in small amounts by the adrenal glands. Testosterone plays an essential role in male sex maturation and spermatogenesis. Interestingly, sex differences in gut microbiota were reduced between emasculated male and female mice (Qin et al., 2010; Yurkovetskiy et al., 2013). Germ-free and SPF mice had differences in serum testosterone levels (Markle et al., 2013). Gonadectomy and hormone replacement significantly affected rodent intestinal microbiota composition (Org et al., 2016). These suggest that testosterone may interact with the gut microbiome.

The synthesis and secretion of testosterone in males vary with gonadal development. It has been reported that the plasma concentration of testosterone gradually increases during pubertal development and reaches peak level at sexual maturation in pigs (Allrich et al., 1983; Schwarzenberger et al., 1993). However, whether such developmental changes of testosterone affect intestinal microbial structure and function is yet to be being determined. Meishan pig is known as an early maturation breed, and the plasma testosterone level positively increases with age, even after sexual maturation (Kanematsu et al., 2006). Therefore, this study investigated the changes in the gut microbiome composition from 10 wk. to 22 wk. in Meishan male pigs, when the plasma testosterone concentration nearly increased twice (Kanematsu et al., 2006). Further, vitro experiments were conducted to verify the effect of testosterone on the gut microbiome. The discoveries will establish a foundation for understanding the interaction of testosterone and the gut microbiota in males.

## 2 Materials and methods

### 2.1 Animals and sampling

This experiment was approved by the Institutional Animal Care and Use Committee (IACUC) of the Shanghai Academy of Agricultural Sciences (Grant number: SAASPZ0520013). All animal care and experimental protocols followed the guidelines of the Laboratory Animal Guideline for Ethical Review of Animal Welfare (GB/T 35892-2018) set by the Standardization Administration of China.

A total of 20 healthy Meishan male pigs were investigated in this study, and all animals were given the same basal diet throughout the whole experiment. Fresh fecal samples were individually collected via rectal massage without contamination with the barn floor at 10 and 22 weeks (wk) of age, respectively. Each fecal sample was mixed homogeneously individually over ice and distributed for different analyses. The fecal sample for microbiome pyrosequencing analysis was filled in a tube with ethanol (1:3, v/v) and stored at −20°C until DNA extraction. The fecal sample for hormone analysis was stored at −20°C.

### 2.2 Determination of fecal testosterone concentration

Compared with blood sampling, fecal sample collection is non-invasive with less interference from acute stress (Sheriff et al., 2010). Fecal steroid hormones concentration can represent the systemic steroid hormones synthesis and secretion, especially for periodic investigation, and now it is commonly used and accepted as a means to study the changes of hormones in domestic and wild animals (Fujita et al., 2001; Sheriff et al., 2010; SankarGanesh et al., 2014). The fecal sample extraction and testosterone measurement followed the protocol described previously (Fujita et al., 2001). In brief, fecal samples were frozen-dried for 72 h, the sample weight before and after frozen-dried was recorded for the calculation of stool water content, and then 100 mg dried fecal powder was extracted in 3 mL of 80% aqueous methanol by vortex for 15 min. Following extraction, we centrifuged the suspension and recovered the supernatant liquid for testosterone measurement. The fecal testosterone was measured by using commercial enzyme-linked immunoassay (EIA) kits of testosterone (Cayman, Item No. 582701, inter-assay CV < 10.7%, intra-assay CV < 14.0%).

### 2.3 Anaerobic culture experiment of fecal bacteria *in vitro*

Fresh feces from 5 experimental castration boars were collected via rectal massage without contamination with the barn floor. In the laboratory, the feces were mixed evenly and sterilized with physiological saline at 1:9 (mass: volume). The diluted material was then homogenized and strained through a four-layer of cheesecloth. After centrifuging at 1,500 rpm for 15 min, the supernatant was taken for inoculation.

The medium for *in vitro* anaerobic culture was modified from that of Williams et al. (2005). The medium contained 4.0 g glucose, 1.0 g trypticase, 1.5 g Pipes, 0.6 g KCl, 0.6 g NaCl, 0.2 g CaCl_2_·2H_2_O, 0.5 g MgSO_4_·7H_2_O, 0.54 g NH_4_Cl, 1.0 mg resazurin solution, 4.1 g Na_2_CO_3_, 1.0 mg Haemin, 2.5 mg NiCl_2_·6H_2_O, 2.5 mg H_3_BO_3_, 2.5 mg Na_2_MoO_4_·2H_2_O, 0.5 mg SeO_2_, 0.5 mg CoCl_2_·6H_2_O, 0.314 mg NaVO_3_, 0.25 mg MnCl_2_·4H_2_O, 0.25 mg ZnCl_2_, 0.25 mg CuCl·2H_2_O, 0.2 mg FeSO_4_·7H_2_O, 68.5 μL acetic acid, 30 μL propionic, 18.4 μL butyric, 5.5 μL valeric, 5.5 μL 2-methyl-butyric, 4.7 μL iso-butyric, and 5.5 μL iso-valeric acids. The solution was filtered and sterilized by 0.22 μm.

The reducing agent contained 20.5 g Na_2_S·9H_2_O and 20.5 cysteines HCl dissolved in 1 L of boiled distilled water with nitrogen gas bubbling through it. This procedure was completed in a fume cupboard due to the danger of inhalation of toxic fumes. The vitamin/phosphate solution contained 0.0204 g biotin, 0.0205 g folic acid, 0.1640 g calcium D-pantothenate, 0.1640 g nicotinamide, 0.1640 g riboflavin, 0.1640 g thiamin HCl, 0.1640 g pyridoxine HCl, 0.0204 g para-aminobenzoic acid, 0.0205 g cyanocobalamin, dissolved in a 1 L of solution containing 54.7 g KH_2_PO_4_. This solution was then filter-sterilized into sterile bottles.

The anaerobic culture trial of fecal bacteria *in vitro* was designed with single factor variance, and the final concentrations of testosterone in the treatments were 200 μg/mL, 400 μg/mL, 800 μg/mL, and 1,200 μg/mL (The solvent for testosterone is DMSO). At the same time, the control group contained the same amount of DMSO as the treatments. Each treatment had six replicates, and each replicate had an 80 mL medium. After 10 min of carbon dioxide flushing, 1 mL reducing agent, 1 mL vitamin-phosphate solution, and 5 mL inoculant were added. After that, the culture was incubated in a shaker at 37°C and 180 rpm for 8 h.

### 2.4 Bacteria pyrosequencing analysis

The total DNA of the fecal microbial community was extracted from individual fecal samples and *in vitro* culture samples, as described previously (Jiang et al., 2020). Illumina Miseq PE250 sequencing was performed based on the bacterial 16S rRNA sequence V3-V4. The data were analyzed on the online platform of Majorbio Cloud Platform.^1^ The operational taxonomic units (OTU) picking with a 97% similarity cut-off was compiled with Qiime using default parameters. Taxonomic classification was performed based on the OTU database. The alpha-diversity indices, including Chao, Ace, Shannon, and Simpson, were calculated with the Mothur program^2^ and Rarefaction software. The community difference at 10 wk. and 22 wk. was evaluated using Principal Co-ordinates Analysis (PCoA) and Adonis test, and a significant difference was assigned at *p* < 0.05.

### 2.5 Statistical analysis

The statistical analyses were performed using SPSS 17.0 for Windows. Independent-Samples *T*-test was used to analyze the significant differences in different treatments. The results were presented as the mean ± standard error of the mean (SEM). Statistical significance was considered at *p* ≤ 0.05 for all analyses. Graph Pad Prism Version 5.01 software (Graph Pad Software, Inc., La Jolla, CA, United States) was used for mapping.

## 3 Results

### 3.1 Experiment *in vivo*

#### 3.1.1 Testosterone concentration

**Figure 1:**
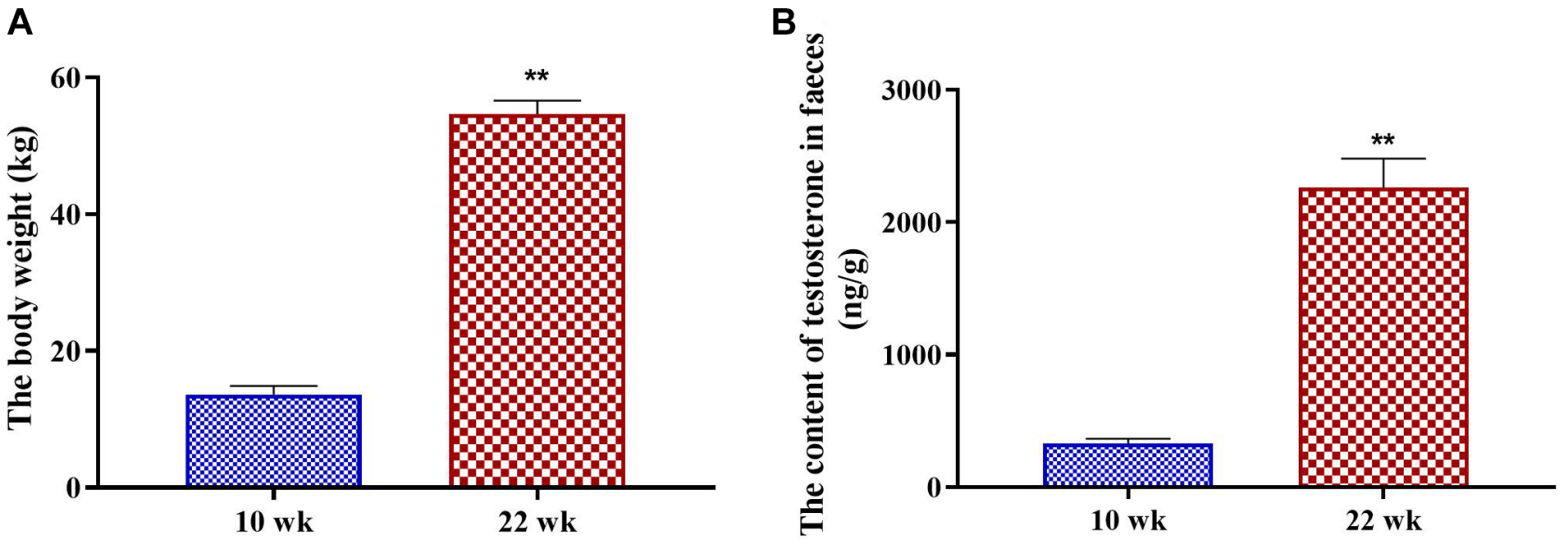
The developmental changes in male pigs. **(A)** The body weight; **(B)** the fecal testosterone content. Graph shows mean value ± SEM (*N* = 20). ** (*p* < 0.01) means significant difference between the males at the age of 10 weeks (10 wk) and 22 weeks (22 wk).

As the male pigs grew from 10 wk. to 22 wk., the body weight increased significantly (*p* = 0.00, Figure 1A). Meanwhile, the fecal testosterone concentration of the male pigs increased significantly from 10 wk. to 22 wk. (*p* = 0.00, Figure 1B).

#### 3.1.2 Fecal microbiota analysis

**Figure 2:**
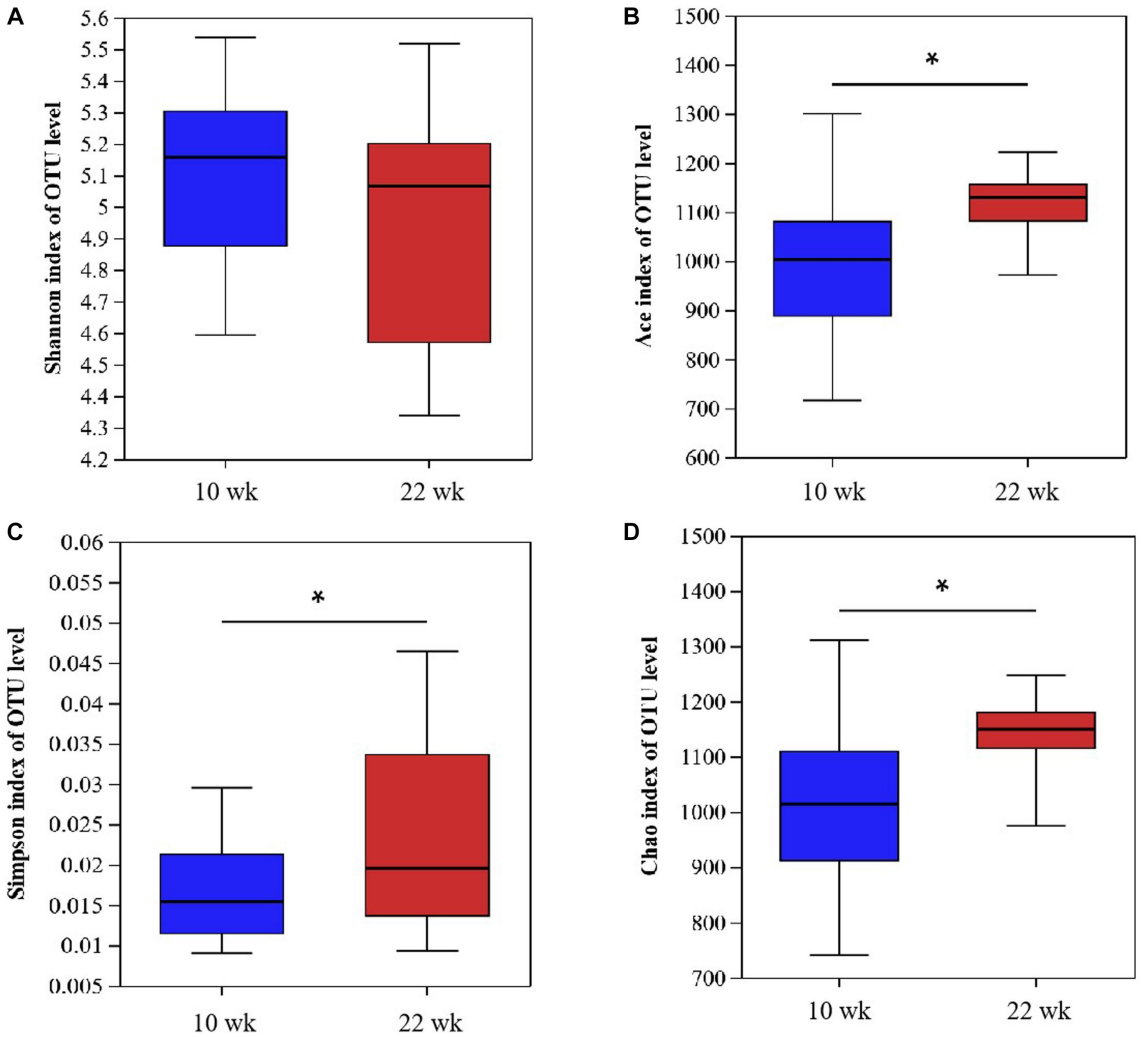
Fecal bacterial richness and alpha diversity index. **(A)** Shannon; **(B)** Ace; **(C)** Simpson; **(D)** Chao. *(*p* < 0.05) means significant difference between the males at the age of 10 weeks (10 wk) and 22 weeks (22 wk). *N* = 20.

**Figure 3:**
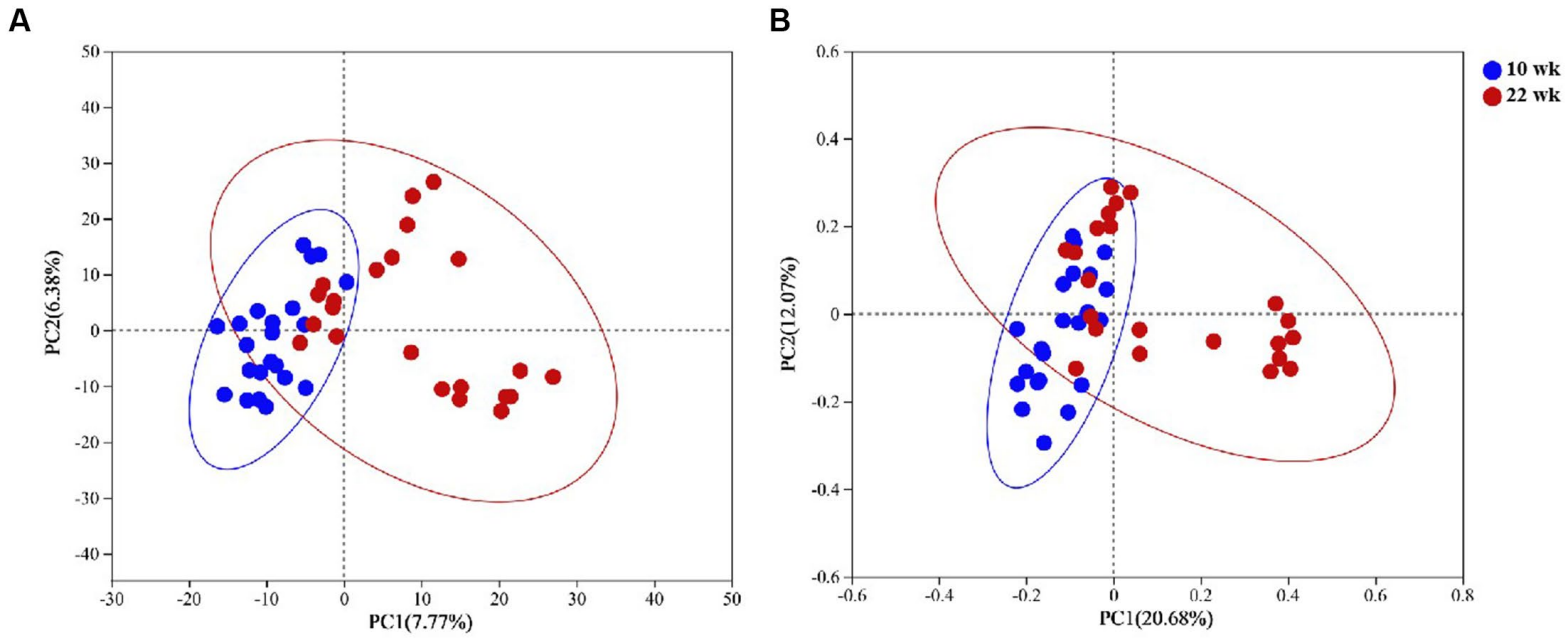
Principal Component Analysis (PCA, **A**) and Principal Co-ordinates Analysis (PCoA, **B**) plots on OTU level. Adonis test showed sexual development time has a significant impact on the fecal microbial community. **(A)** Based on Bray-Curtis distance and *R*^2^ = 0.09, *p* = 0.00; **(B)** Based on weighted unifrac distance and *R*^2^ = 0.12, *p* = 0.00 at the OTU level. Red dots represent the male pigs at 10 weeks (10 wk), blue triangles represent the male pigs at 22 weeks (22). *N* = 20.

The 16S ribosomal RNA gene sequencing obtained 2,251,025 high-quality sequencing reads from 40 samples. Based on 97% species similarity, 1815 OTUs were obtained, which involved 476 different OTU classifications. The bacterial DNA pyrosequencing profile (Figure 2) observed that the Ace, Chao, and Simpson were higher at 22 wk. than at 10 wk. (*p* < 0.05). Principal Component Analysis (PCA)(a) and Principal Co-ordinates Analysis (PCoA) visually confirmed that the fecal microbial communities at 22 wk. distinctly separated from those at 10 wk. (Figure 3).

**Figure 4:**
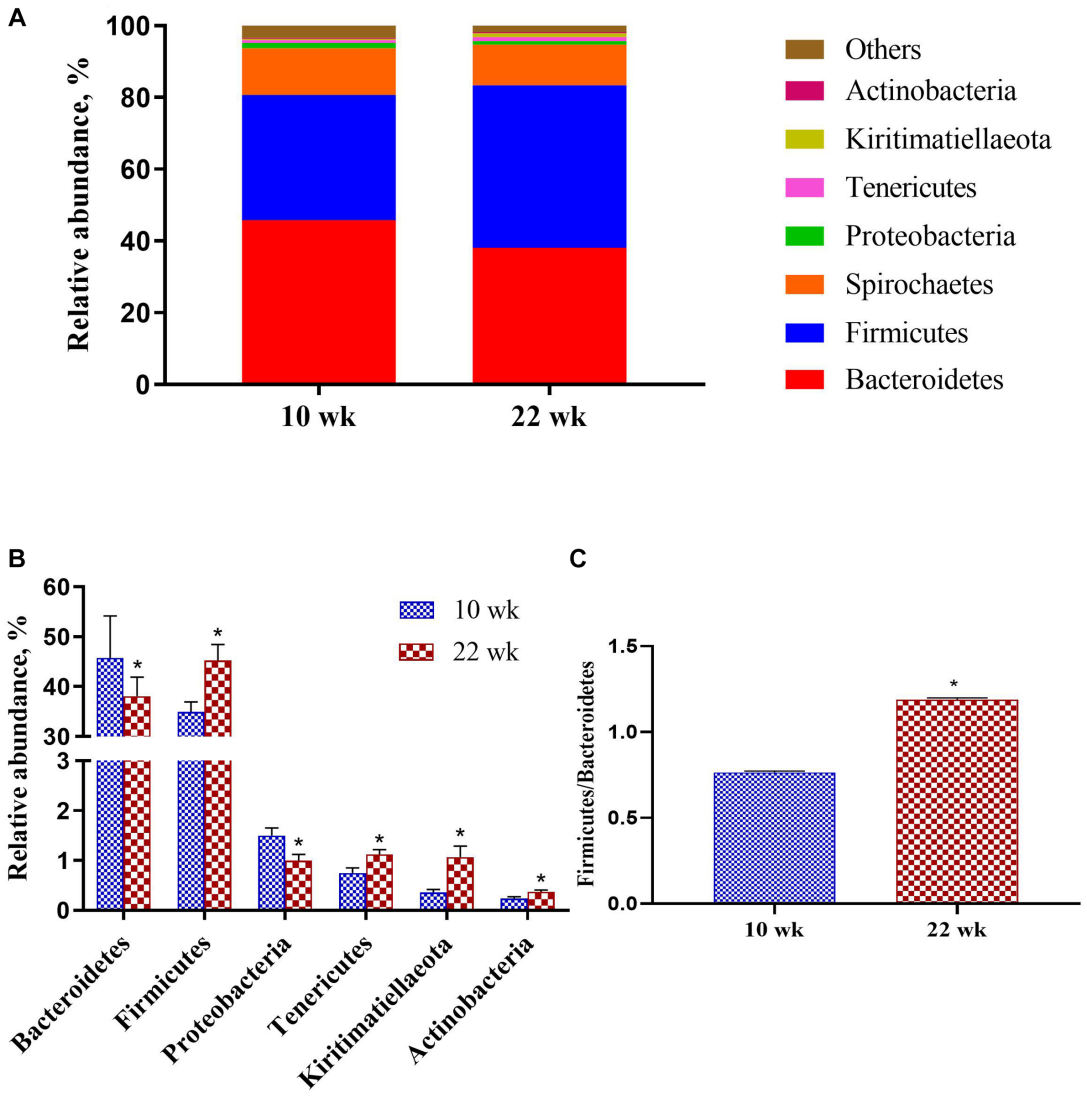
Taxonomic classification of the 16S rRNA gene sequences at the phylum level. **(A)** Fecal microbiota composition; **(B)** Composition difference; **(C)** the *Firmicutes/Bacteroidetes* ratio. Graph shows means value ± SEM (*N* = 20). * (*p* < 0.05) means significant difference between the males at the age of 10 weeks (10 wk) and 22 weeks (22 wk).

At the phylum level (Figure 4A), the majority proportions of sequences attributed to *Bacteroidetes* (>41.94%) and *Firmicutes* (>40.12%). Following the growth of the pigs from 10 wk. to 22 wk. (Figure 4B), there was a significant reduction of *Bacteroidetes* (*p* = 0.02) and *Proteobacteria* (*p* = 0.01) but a pronounced increase of *Firmicutes* (*p* = 0.02), *Tenericutes* (*p* = 0.01), *Kiritimatiellaeota* (*p* = 0.01) and *Actinobacteria* (*p* = 0.02). Moreover, the *Firmicutes* / *Bacteroidetes* ratio at 10 wk. was significantly lower than at 22 wk. (*p* = 0.02, Figure 4C).

**Figure 5:**
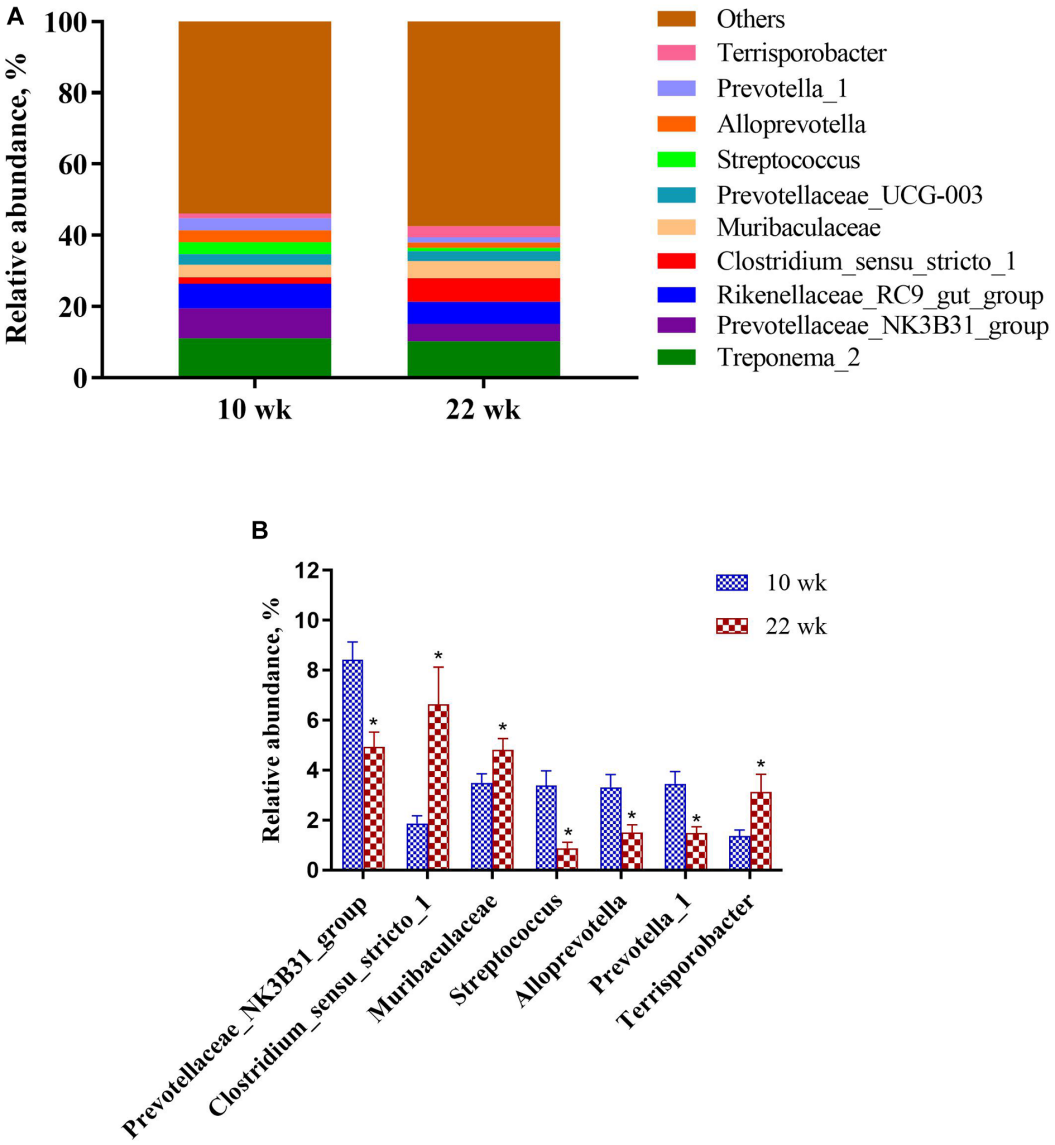
The column chart identifying the significantly different taxa between the different age at the genus level. **(A)** Gut microbiota composition; **(B)** Composition difference. Graph shows means value ± SEM (*N* = 20). * (*p* < 0.05) means significant difference between the males at the age of 10 weeks (10 wk) and 22 weeks (22 wk).

At the genus level (Figure 5), the proportions of *Prevotellaceae_NK3B31_group* (*p* = 0.00), *Streptococcus* (*p* = 0.00), *Alloprevotella* (*p* = 0.01), and *Prevotella_1* (*p* = 0.00) of the fecal microbial composition were significantly lower at 22 wk. than that at 10 wk., while *Clostridium_sensu_stricto_1* (*p* = 0.00), *Muribaculaceae* (*p* = 0.02) and *Terrisporobacter* (*p* = 0.03) were higher at 22 wk. than that at 10 wk.

**Figure 6:**
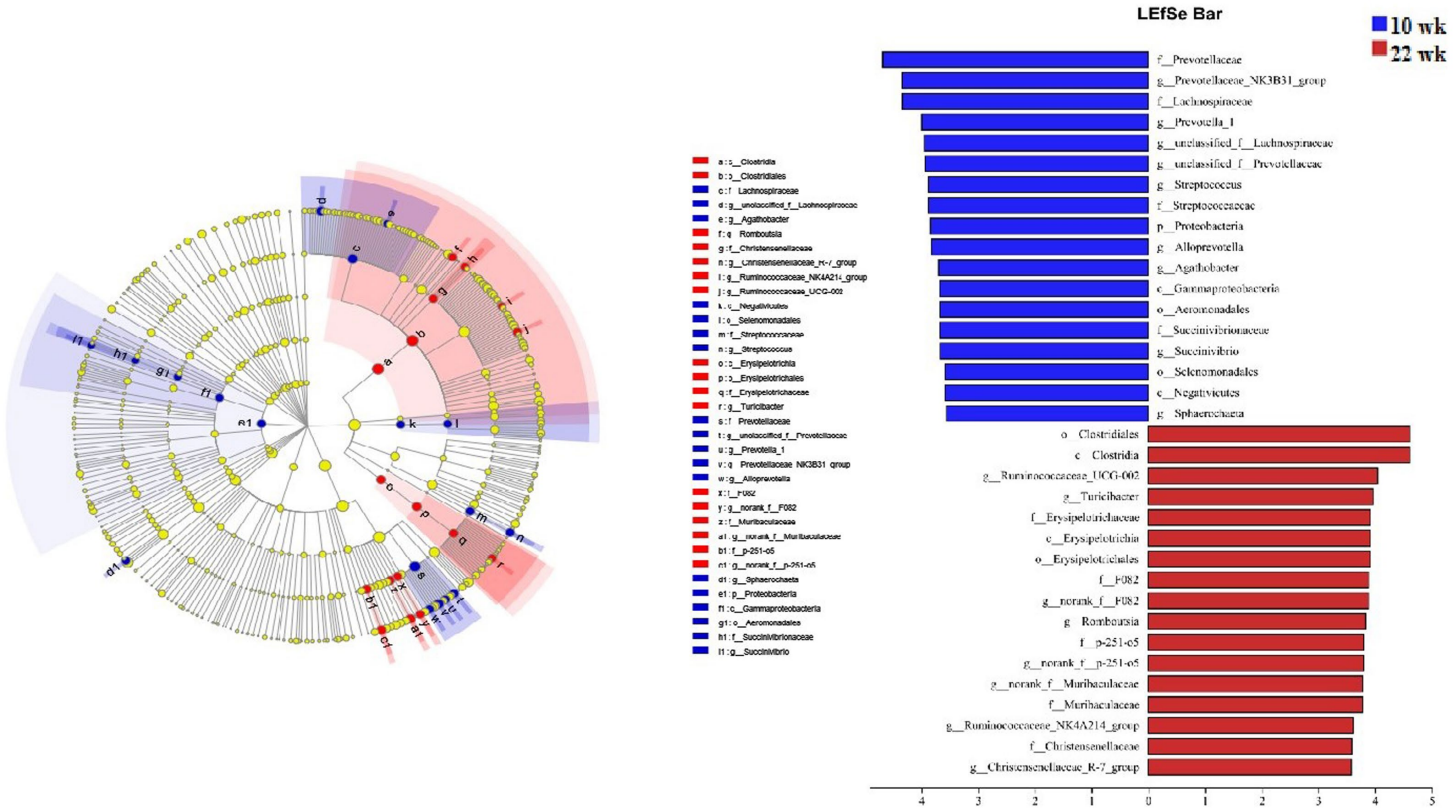
Linear Discriminant Analysis Effect Size (LEfSe) of the most dominant bacteria in the fecal microbiota of the males at the age of 10 weeks (10 wk) (red) and 22 weeks (22 wk) (blue) at genus level (*N* = 20; all linear discriminant analysis [LDA] scores (log10) >3.5).

Additionally, LEfSe analysis was performed to determine the abundance of specific microbial taxa between the male pigs at 10 wk. and 22 wk. (Figure 6). An LDA score (log10) greater than 3.5 was considered the threshold. The genera *Prevotellaceae_NK3B31_group*, *Prevotella_1*, *Prevotellaceae*, *Streptococcus*, *Lachnospiraceae*, *Alloprevotella*, *Agathobacter*, *Sphaerochaeta*, and *Succinivibrio* enriched at 10 wk., and the genera *Ruminococcaceae_UCG-002*, *Turicibacter*, *Romboutsia*, *Muribaculaceae*, *Ruminococcaceae_NK4A214_group*, *Christensenellaceae_R-7_group* and *Lachnospiraceae_XPB1014_group* improved at 22 wk.

#### 3.1.3 Correlation analysis between testosterone level and fecal differential microbe

**Figure 7:**
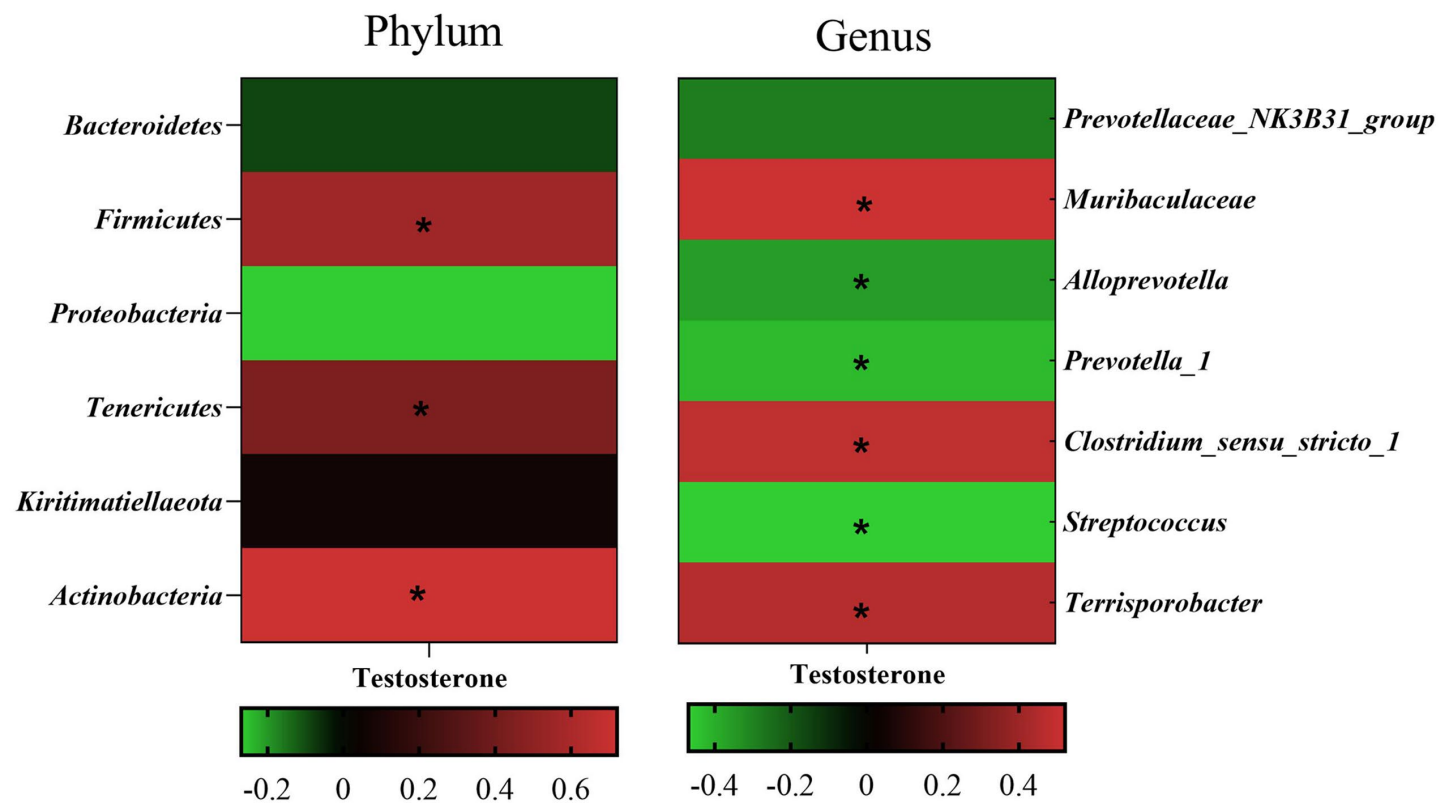
Pearson correlation analysis between fecal testosterone level and differential microbe at phylum level and genus level. The cells are colored based on the Pearson correlation coefficient between the testosterone level and differential microbe between different age, red represents positive correlation, green represents negative correlation, and * represents that the correlation was significant (*p* < 0.05).

Pearson’s correlation coefficient analysis (Figure 7) observed that the fecal testosterone level was significantly positively associated with the relative abundance of phylum *Firmicutes* (*r* = 0.56, *p* = 0.00), *Tenericutes* (*r* = 0.44, *p* = 0.00), and *Actinobacteria* (*r* = 0.72, *p* = 0.00), and genus *Muribaculaceae* (*r* = 0.52, *p* = 0.00), *Clostridium_sensu_stricto_1* (*r* = 0.48, *p* = 0.00). However, the fecal testosterone level was significantly negatively associated with the relative abundance of genus *Alloprevotella* (*r* = −0.36, *p* = 0.02), *Prevotella_1* (*r* = −0.43, *p* = 0.00), and *Streptococcus* (*r* = −0.47, *p* = 0.00).

### 3.2 Experiment *in vitro*

#### 3.2.1 Depletion rate of testosterone in the microbial communities culture medium

**Figure 8:**
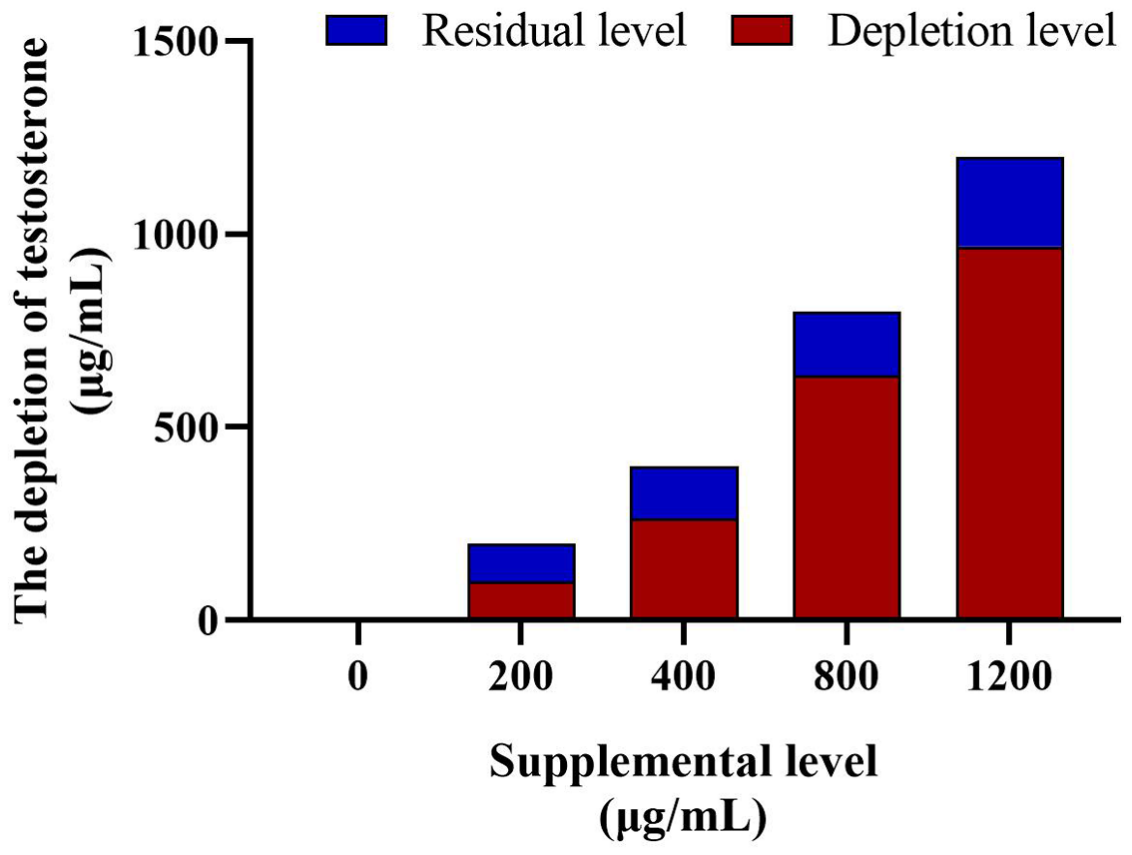
The testosterone depletion in culture medium among the different testosterone treatment group. The depletion rates of testosterone are 60.27, 66.20, 71.37%, and 78.23 in turn. *N* = 6.

The EIA assay determined the testosterone concentration in the culture solution and did not observe testosterone in the control group. However, the testosterone levels in the culture medium of the testosterone treatments (200 μg/mL, 400 μg/mL, 800 μg/mL, and 1,200 μg/mL) decreased significantly (*p* < 0.05) after 8 h culture, the depletion rate inclined with the increase of supplemental testosterone level (50.76 ± 2.61%, 66.20 ± 2.63%, 79.24 ± 1.14%, and 80.68 ± 1.01%, respectively, Figure 8).

#### 3.2.2 Changes in bacterial diversity

**Figure 9:**
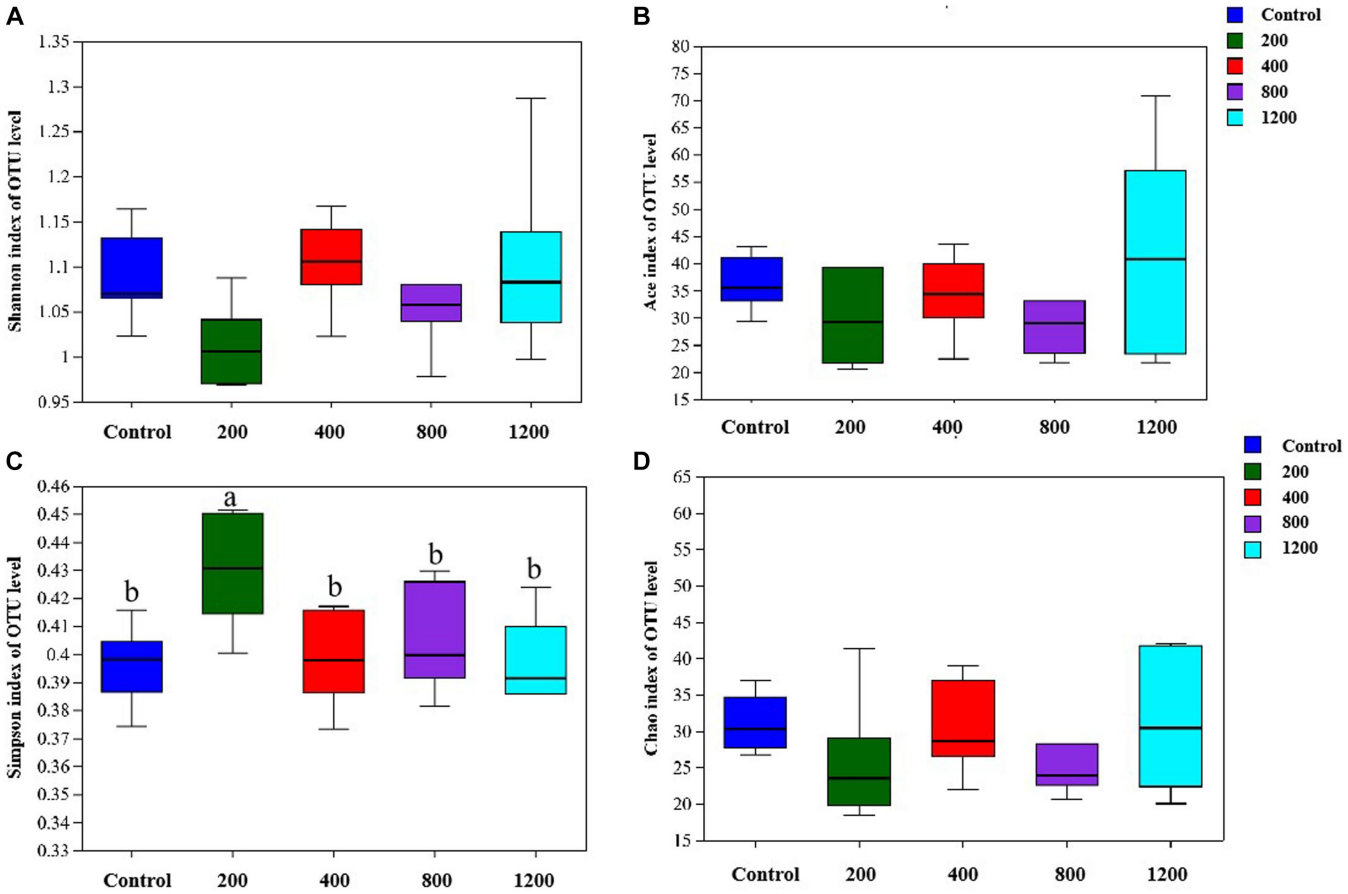
The richness and alpha diversity index of the culture microbial communities. **(A)** Shannon; **(B)** Ace; **(C)** Simpson; **(D)** Chao. Graph shows means value ± SEM (*N* = 6), and the bars with different letters indicate significant difference (*p* < 0.05).

The bacterial DNA pyrosequencing profile (Figure 9) observed that testosterone had no significant effect on richness estimators (Ace and Chao) of microbial communities of the *in vitro* culture. Nevertheless, the 400 μg/mL testosterone treatment achieved the most extensive Shannon index, higher than the 200 μg/mL testosterone and 1,200 μg/mL testosterone treatment (*p* < 0.05). Moreover, the Simpson of the 200 μg/mL testosterone treatment was significantly higher than other treatments (*p* < 0.05).

**Figure 10:**
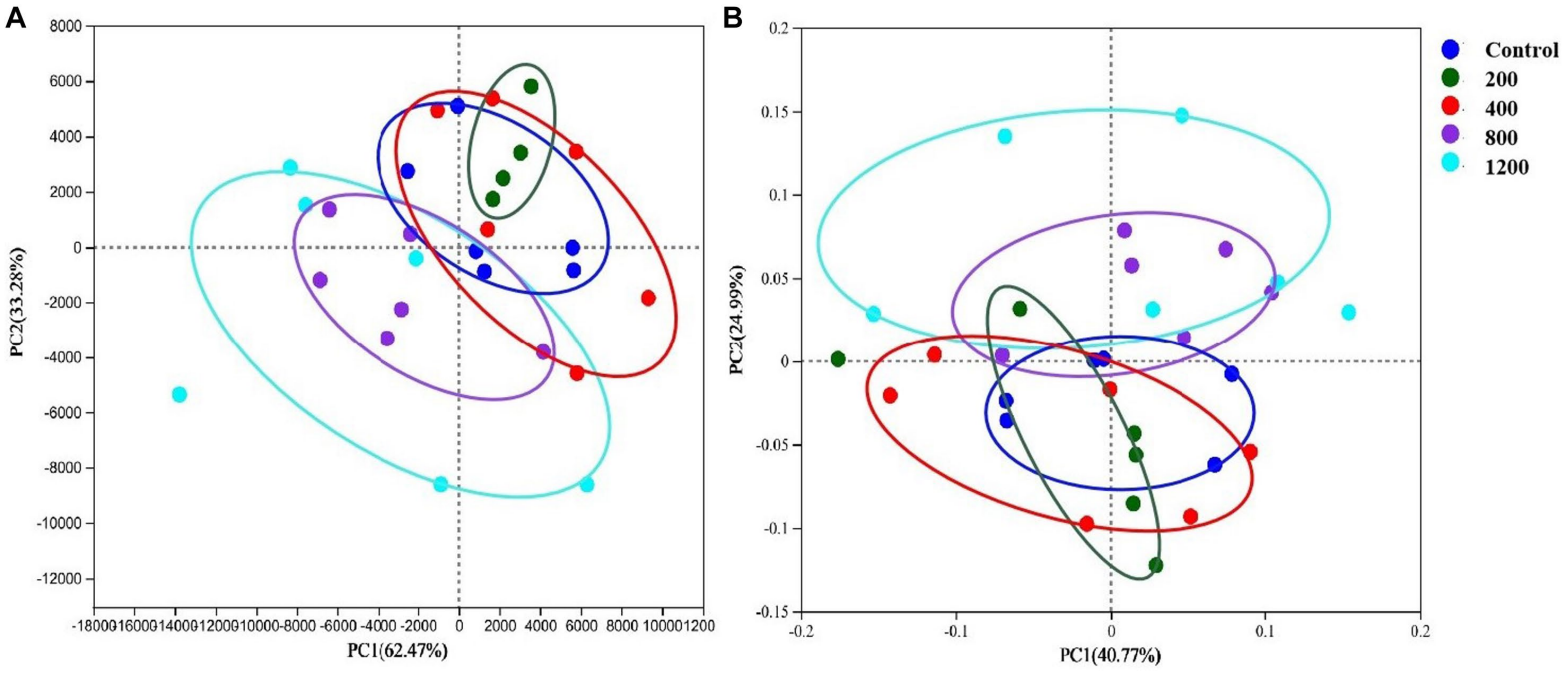
Principal Component Analysis (PCA, **A**) and Principal Co-ordinates Analysis (PCoA, **B**) plots on OTU level. Adonis test showed testosterone has a significant impact on the microbial community. **(A)** *R*^2^ = 0.37, *p* = 0.00; **(B)** *R*^2^ = 0.27, *p* = 0.03 at the OTU level. *N* = 6.

Although there was no clear visual separation of groups within the testosterone treatments in Figure 10, the inter-group variation was higher than the inter-individual variation of the treatment. The PCA (Figure 10A) and PCoA (Figure 10B) visually confirmed the distinct separation of microbial communities at the OTU level (*p* = 0.00 and *p* = 0.03) among the five groups.

#### 3.2.3 Microbiota analysis

**Figure 11:**
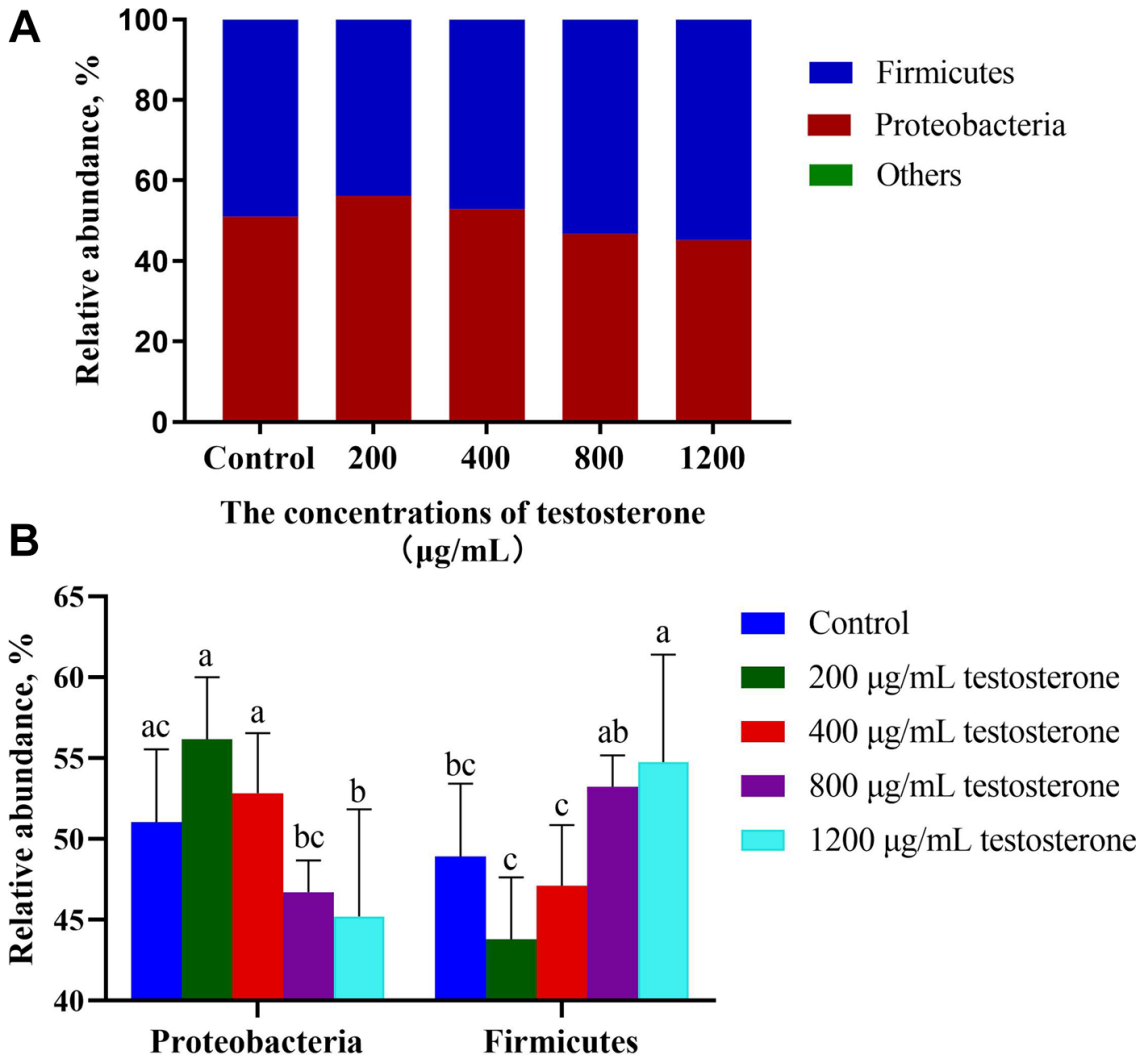
Taxonomic classification of the 16S rRNA gene sequences at the phylum level for the testosterone treatment. **(A)** Gut microbiota composition; **(B)** Composition difference. Graph shows means value ± SEM (*N* = 6), and the bars with different letters indicate significant difference (*p* < 0.05).

The *in vitro* culture trial observed that *Proteobacteria* and *Firmicutes* were the dominant phylum (Figure 11A). The abundance of *Proteobacteria* in the 800 and 1,200 μg/mL testosterone treatments was significantly lower than in the 200 and 400 μg/mL testosterone treatments (*p* < 0.05, Figure 11B). The abundance of *Firmicutes* in the 800 and 1,200 μg/mL testosterone treatments was significantly higher than the 200 and 400 μg/mL testosterone (*p* < 0.05, Figure 11B).

**Figure 12:**
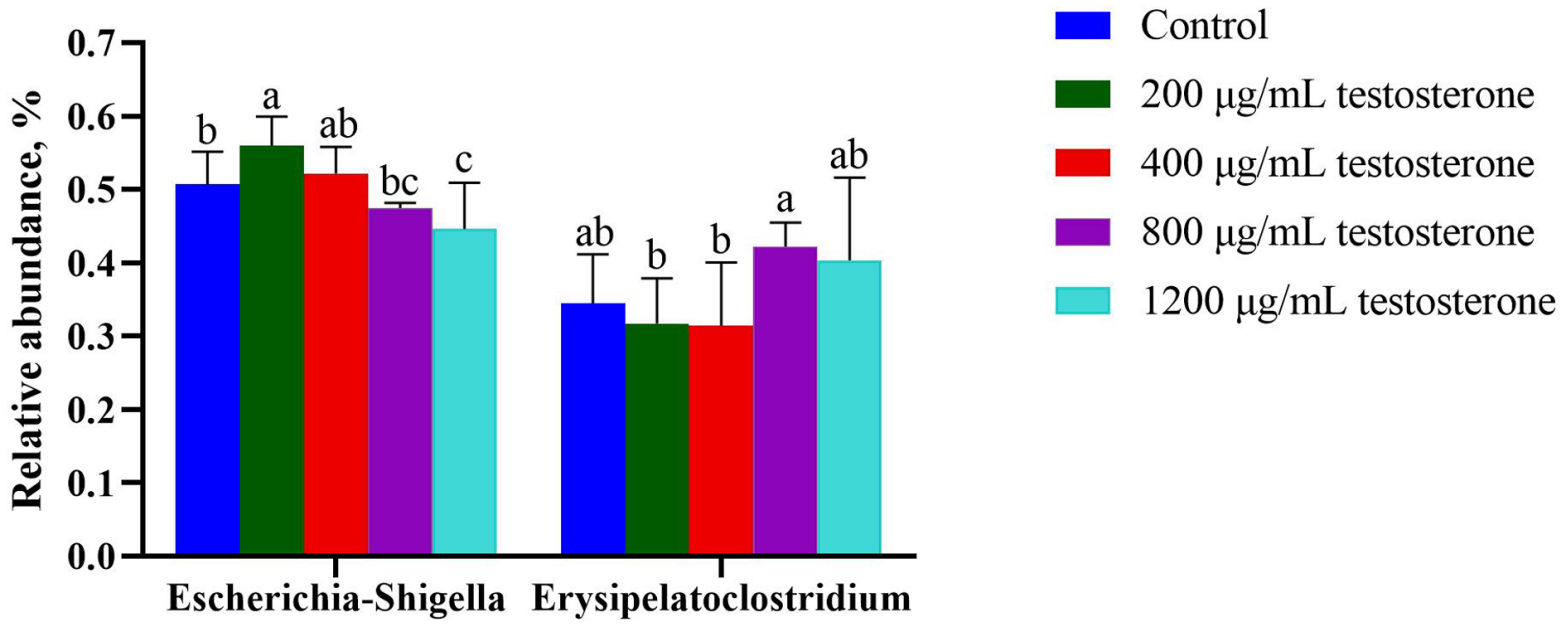
The column chart identifying the significantly different taxa among the testosterone treatment group at the genus level. Graph shows means value ± SEM (*N* = 6), and the bars with different letters indicate significant difference (*p* < 0.05).

At the genus level (Figure 12), with the increase in supplemental testosterone level, the proportion of *Escherichia-Shigella* decreased. In contrast, the abundance of *Erysipelatoclostridium* in the 800 μg/mL testosterone treatment was higher than the 200 and 400 μg/mL testosterone (*p =* 0.04 and *p =* 0.03).

## 4 Discussion

The present study revealed the relationship between testosterone and the composition of intestinal microorganisms during the gonad development of Meishan male pigs. It is well known that testosterone has a significant effect on the physiology and behavior of animals. Still, it has been more challenging to show that testosterone differences lead to differences in the gut microbiome of animals. In the present study, male Meishan pigs had higher fecal testosterone at 22 wk. of age than 10 wk. of age, which is consistent with previous studies that the plasma testosterone level in male Meishan pigs increased gradually from birth to 30 wk. of age and positively correlated with the bodyweight development (Kanematsu et al., 2006). Meanwhile, the fecal microbiome had higher Ace, Chao, and Simpson at 22 wk. of age than that at 10 wk., and the microbial communities at 22 wk. of age grouped differently from 10 wk. in the PCA and PCoA. Furthermore, *in vitro* trials confirmed that the different testosterone treatments (0, 200, 400, 800, 1,200 μg/mL) had distinct separation of microbial communities at the OTU level of PCoA. The present study via *in vivo* and *in vitro* trials showed that the intestinal microbial composition changed with testosterone levels.

As previously reported, *Firmicutes* and *Bacteroidetes* dominated the fecal microbiota at the phylum level (Xiao et al., 2017; Wang H. et al., 2019; Guo et al., 2020). The *Firmicutes* to *Bacteroidetes* ratio was relevant in signaling human gut microbiota status (Ley et al., 2006). It can be linked to overall changes in bacterial profiles at different stages of life (Mariat et al., 2009). The study of Mariat et al. (2009) also showed that the *Firmicutes* to *Bacteroidetes* ratio undergoes an increase from birth to adulthood. Consistently, the *Firmicutes* to *Bacteroidetes* ratio was higher at 22 wk. of male pigs, and the abundance of phylum *Firmicutes* positively correlated with testosterone content. Furthermore, the *in vitro* trial indicated that the mass of *Firmicutes* increased parallel to the increase in testosterone dosage. These suggest that the development of testosterone secretion may be attributed to changes in the fecal microbiota associated with advancing age.

The phylum *Proteobacteria* includes several gastrointestinal pathogens, such as diarrheagenic *Escherichia coli* (Cordonnier et al., 2016; Wang X. et al., 2019). Morgan et al. (2012) observed expansive *Proteobacteria* in intestinal inflammation, including irritable bowel syndrome and metabolic syndrome. Moreover, high abundances, or ‘blooms’ of *Proteobacteria* in the gastrointestinal tract, have been suggested as a microbial signature of dysbiosis in mammals (Shin et al., 2015). Numerous studies indicated that the abnormal expansion of *Proteobacteria* is a potential diagnostic microbial characteristic of gut microbiota imbalance and epithelial dysfunction (Litvak et al., 2017). *Streptococcus* is an early colonizer in the gut of pigs, and an increase in the abundance of *Streptococcus* may also initiate infection. Here, as male pigs developed from 10 wk. to 22 wk., the fecal testosterone level increased, while the proportion of *Proteobacteria* phylum and *Streptococcus* genera decreased. Moreover, the *in vitro* trial indicated that testosterone treatments inhibited *Escherichia-Shigella* growth. These suggest that testosterone may be advantageous for formatting a healthy gut microbial community in male pigs, combined with the developmental changes in the *Firmicutes* to *Bacteroidetes* ratio and fecal testosterone.

In addition, the present study observed that the abundance of the phylum *Actinobacteria*, *Kiritimatiellaeota*, and *Tenericutes*, and genera *Alloprevotella*, *Clostridium_sensu_stricto_1*, *Muribaculaceae*, *Prevotellaceae_NK3B31_group*, *Prevotella_1*, and *Terrisporobacter* differed from 10 wk. to 22 wk. The abundance of phylum *Actinobacteria* and *Tenericutes*, and genera *Clostridium_sensu_stricto_1* and *Muribaculaceae* positively correlated with the fecal testosterone content, whereas genera *Alloprevotella*, *Prevotella_1* negatively correlated with the fecal testosterone level. However, data about the changes in bacteria and testosterone were inconsistent. The phylum *Actinobacteria* is associated with synthesizing antibiotics, immunomodulatory compounds, and metabolites, which are essential for animal health. Niu et al. (2015) observed that the abundance of *Clostridium* and *Tenericutes* in the gut positively correlated with the apparent crude fiber digestibility in pigs. *Muribaculaceae* may be associated with carbohydrate degradation (Lagkouvardos et al., 2019). Zhang et al. (2022) observed that the proportion of *Alloprevotella* and *Muribaculaceae* increased in the gut of piglets under cold stress. Women with Polycystic Ovary Syndrome had a reduced relative abundance of the phylum *Tenericutes* in the fecal microbiome (Lindheim et al., 2017). A clinical study showed that *Alloprevotella* was enriched in obese participants (Insenser et al., 2018). Diethylhexylphthalate-exposed mice had a decrease in blood testosterone levels and a reduction of *Firmicutes* and *Paraprevotella* in the fecal microbiota, while an increase in *Bacteroidetes* and *Prevotella* (Tian et al., 2019). A previous study showed that *Prevotella* could produce succinate and acetate, which could improve the gut barrier and exhibit anti-inflammatory function, the abundance of *Prevotella* decreased from 30% of all bacteria to 4.0% as pigs aged (Xiao et al., 2017). These discrepant data suggest that the relationship between testosterone and gut microbiota may differ in the developmental stage and health status.

While steroid hormones model the gut microbiome composition and diversity, the bacteria may influence testosterone secretion and metabolism. Neuman et al. (2015) reported that transplantation of male microbiome to females caused an increase in the testosterone and enrichment of *E. coli* and *Shigella-like* bacteria in females. *Clostridium* can convert glucocorticoids to androgens, a group of male steroid hormones. Genera *Prevotella* can use estradiol and progesterone as alternative sources for growth (Kornman and Loesche, 1982). The *Firmicutes*, *Proteobacteria*, and *Actinobacteria* can metabolize and degrade steroid hormones (García-Gómez et al., 2013). Fecal bacteria, such as *Bacillus*, *Brevibacterium*, *Streptomyces*, and *Pseudomonas*, could degrade testosterone (Yang et al., 2010; Shih et al., 2017). Present data agreed with the above that the consumption rate of testosterone increased gradually with the treated testosterone dosage in the culture medium, further illustrating that the bacteria could degrade testosterone.

To conclude, the present study found a significant correlation between the change in testosterone content and microflora. The vitro trials showed that testosterone could modulate the microflora structure. Meanwhile, the bacteria could degrade the testosterone in a dose testosterone-dependent manner. Whether such interaction contributes to testosterone and gut microbiome development changes remains unclear and further studies are needed.

## Data availability statement

The datasets presented in this study can be found in online repositories. The names of the repository/repositories and accession number(s) can be found below: Raw read sequences of the 16S rRNA gene from Meishan pig feces-associated microbiota in this study are publicly available in the NCBI SRA depository within BioProject PRJNA998586, with BioSample accession numbers SAMN36707203-SAMN36707246.

## Ethics statement

The animal study was approved by Institutional Animal Care and Use Committee (IACUC) of Shanghai Academy of Agricultural Sciences Shanghai Academy of Agricultural Sciences. The study was conducted in accordance with the local legislation and institutional requirements.

## Author contributions

XJ: Conceptualization, Data curation, Formal Analysis, Project administration, Writing – original draft, Writing – review & editing. SD: Investigation, Methodology, Writing – review & editing. NL: Investigation, Validation, Writing – review & editing. WY: Formal Analysis, Writing – review & editing. DX: Supervision, Writing – review & editing. WT: Formal Analysis, Writing – review & editing. HL: Formal Analysis, Writing – review & editing. PJ: Investigation, Writing – review & editing. YG: Resources, Writing – review & editing.

## Footnotes

^1^http://www.majorbio.com

^2^http://www.mothur.org
